# Effect of the substrate on the electrical transport and fluctuation processes in NbRe and NbReN ultrathin films for superconducting electronics applications

**DOI:** 10.1038/s41598-022-05511-5

**Published:** 2022-01-28

**Authors:** C. Barone, C. Cirillo, G. Carapella, V. Granata, D. Santoro, C. Attanasio, S. Pagano

**Affiliations:** 1 Dipartimento di Fisica “E.R. Caianiello”, Università degli Studi di Salerno, 84084 Fisciano, Salerno Italy; 2 CNR-SPIN, c/o Università degli Studi di Salerno, 84084 Fisciano, Salerno Italy; 3 INFN Gruppo Collegato di Salerno, c/o Università degli Studi di Salerno, 84084 Fisciano, Salerno Italy

**Keywords:** Materials science, Electronic properties and materials, Superconducting properties and materials, Surfaces, interfaces and thin films

## Abstract

NbRe-based superconducting thin films recently received relevant interest in the field of low-temperature electronics. However, for these materials the electrical conduction mechanisms, in particular in the normal state, still need to be investigated in more detail. Here, NbRe and NbReN films of different thicknesses have been deposited on two different substrates, namely monocrystalline Si and $\text {SiO}_2$ buffered Si. The films were characterized by DC electrical transport measurements. Moreover, a connection with the charge carriers fluctuation processes has been made by analyzing the electrical noise generated in the normal state region. Despite the films morphology seems not to be affected by the substrate used, a lower noise level has been found for the ones grown on $\text {SiO}_2$, in particular for NbReN. From this study it emerges that both NbRe and NbReN ultrathin films are of very good quality, as far as the low-temperature electrical noise and conduction are concerned, with noise levels competitive with NbN. These results may further support the proposal of using these materials in a nanowire form in the field of superconducting electronics.

## Introduction

Low-temperature electronics is a broad and prosperous field of research, which comprises the study and realization, among the others, of devices for quantum communication and computation, spintronics, space electronics and sensing^1,2^. Superconducting Nanowire Single-Photon Detectors (SNSPDs) are the core of many low-temperature devices for a wide range of applications^3,4^. Their versatility is the reason which makes researchers constantly working for improving their already brilliant performances. In particular, the choice of the superconducting material on which they are based is crucial in determining their properties^5^.

Recently, NbRe-based superconducting films were proposed for the realization of fast SNSPDs, namely NbRe^6,7^ and NbReN^8^. In this respect, a detailed characterization of the films is useful to clarify if these two materials may indeed represent a solid alternative to the other well-established superconductors used in this field, such as NbN^9^, NbTiN^10^, or amorphous WSi^11^. While the first single photon detectors made of NbRe were already realized and showed good metrics, especially concerning time performances^12^, NbReN still need to be tested in a device form, although NbReN-based SNSPDs are expected to extend the operational frequency range of NbRe with comparable time response. This is due to the reduced value of the superconducting gap, comparable to that of amorphous superconductors, and to the short quasi-particle relaxation rates^8^.

With the aim of gaining a deeper insight into the properties of both NbRe and NbReN films, we performed different experimental investigations, including morphological, noise and DC electrical transport measurements, mainly in the normal state, on films of different thickness. In particular, morphological and noise measurements, that were not reported so far for these materials, may be of great interest. As far as the films structure is concerned, X-ray diffraction revealed that the films are polycrystalline with grains of small dimensions and moderate texture^6,8^. For applications as SNSPD, a layer made of small crystallites may reduce the issues related to constrictions and structural defects and in principle does not set strict requirements on the substrate structure and dimensions and simplifies the patterning procedures. For these reasons we have analyzed the films surfaces by atomic force microscopy (AFM) to gain additional information about the samples uniformity, roughness and crystallinity. On the other hand, the study of the charge carriers fluctuation processes provides a deeper comprehension of the physical mechanisms in a large variety of systems, as observed in iron-based superconducting materials^13,14^ and nanowires^15^, and in granular aluminum oxide superconducting thin films^16,17^ and nanostructures^18^, as well as in superconducting qubits^19^. Fluctuation phenomena may influence the main figures of merit of the SNSPD devices, such as jitter^20^, dark counts^21,22^, and latching^23^. Moreover, their study may help revealing details about the detection mechanism in single photon detectors. As an example, it was recently demonstrated the role played by Fano fluctuations in determining the detection efficiency and the timing jitter^24^. Indeed, the operation of a photon detector is intrinsically based on nonequilibrium dynamics^25^, which may be strongly affected by statistical fluctuations phenomena. For instance, the interaction between the quasi-particles (qp) and the substrate via phonon escape in the last stage of the detection process, is an example of normal state processes where fluctuations may play an important role^21^.

## Experimental results and discussion

### Morphological properties

**Figure 1:**
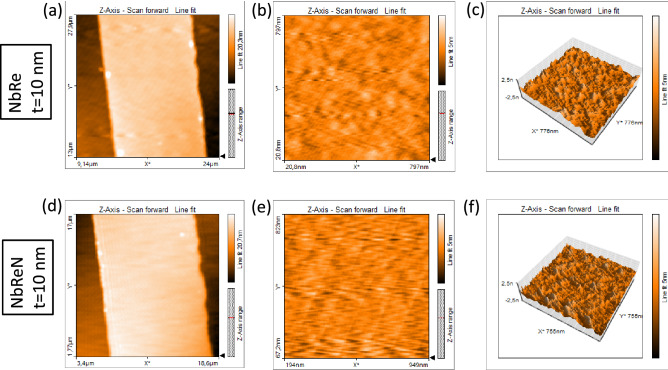
Atomic force microscope (AFM) images. Large area, small area, and three-dimensional scans for typical 10-nm-thick NbRe (**a**–**c**) and NbReN (**d**–**f**) films, respectively, deposited on Si substrates. Very similar results are obtained by using $\text {SiO}_{{2}}$ substrates.

The morphology and roughness of the film surface strongly affect its transport properties, such as, for instance, the value of the resistivity. In the case of NbRe and NbReN the structural characterization revealed that films consist of small crystallites, which are responsible of disorder-dominated electrical conduction^6,8^. The AFM analysis presented in this work confirms this picture. The results of the AFM measurements are summarized in Fig. 1. In particular, panels (a), (b), and (c) [(d), (e), and (f)] refer to a typical NbRe [NbReN] film 10-nm-thick deposited on Si substrate. The two-dimensional AFM micrographs, acquired on both large [panels (a), (d)] and small [panels (b), (e)] scales, reveal smooth surfaces with no evident grain boundaries, superficial defects or impurities. From the three-dimensional images [panels (c), (f)] it is even more evident that, for both NbRe and NbReN, very small grains uniformly cover the surface of the substrate, producing low-roughness profiles. For the NbRe film, on a 1 squared micron area, a root mean square surface roughness $S_{q} = 0.4$ nm is measured. The surface roughness is slightly lower, $S_{q} = 0.3$ nm, in the case of NbReN. Consequently, both films are found relatively flat, with roughness comparable to that of the used substrates, nominally $S_{q} = 0.3$ nm both for Si and $\text {SiO}_{{2}}$. In summary, all the analyzed samples exhibit very small grain sizes, with apparent diameter smaller than 20 nm even in the case of the films 100-nm-thick. This result confirms that the films under study are moderately oriented with small crystallites in agreement with X-ray diffraction measurements reported in Refs.^6^ and^8^. Here, it is important to underline that similar AFM images are observed independently from the substrate used for the deposition. Therefore, morphological properties seem to be not influenced by the substrate, either Si and $\text {SiO}_{{2}}$. Hereafter, by Si substrate we mean a Boron-doped Si(100) and by $\text {SiO}_{{2}}$ substrate we mean a Boron-doped Si(100) covered by a 200-nm-thick amorphous $\text {SiO}_{{2}}$ layer.

### Electrical transport properties

**Figure 2:**
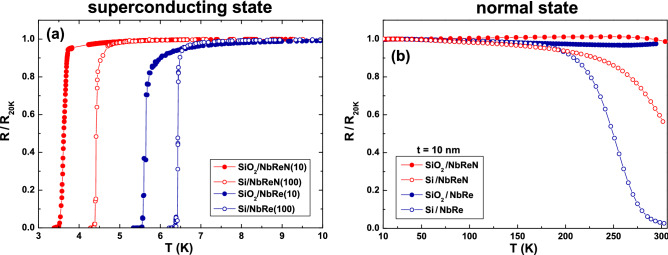
Electrical transport DC measurements. (**a**) Temperature dependence of the normalized resistance, $R/R_{\mathrm {20K}}$, in the superconducting transition region. Blue and red circles refer to NbRe and NbReN films, respectively, while full and open symbols refer to 10-nm-thick (ultrathin) and 100-nm-thick (bulk) ones, respectively. (**b**) Temperature dependence of the normalized resistance, $R/R_{\mathrm {20K}}$, in the normal state region for NbRe and NbReN 10-nm-thick films. Filled and open symbols refer to samples deposited on $\text {SiO}_{{2}}$ and Si substrates, respectively.

The superconducting critical temperature $T_{c}$, along with the critical current density $J_{c}$, are two fundamental parameters whose values must be considered to evaluate the possible applicability of NbRe and NbReN for the realization of SNSPDs. In particular, the $T_{c}$ value is directly related to the minimum photon energy detectable by the device^7^. The low-temperature dependence of the normalized resistance, defined as the ratio of the resistance and its value at $T = 20$ K ($R/R_{\mathrm {20K}}$), of the films under study is reported in Fig. 2a. As evidenced by looking at open symbols (100-nm-thick films) and at full symbols (10-nm-thick films) the critical temperature is depressed as the thickness is reduced. In the case of NbRe (NbReN) the $T_{c}$, defined at the midpoint of the transition, is reduced from 6.44 (4.42) K to 5.64 (3.62) K when passing from 100 to 10 nm. However, it is important to notice, that also the NbReN film 10-nm-thick has a critical temperature easily reachable with a common cryogen-free system. While the deposition parameters strongly affect the transport properties of NbRe and NbReN samples both in the normal and in the superconducting states^6,8^, the $T_{c}$ of the NbReN films deposited on Si and $\text {SiO}_{{2}}$ differ only slightly (see supplementary Fig. S1). Similarly, the choice of the substrate does not influence the values of the transition widths, which are in agreement with those reported in Refs.^6^ and^8^.

Useful information can be extracted by analyzing the normal state DC electric transport properties. The temperature dependence from 300 down to 10 K of the normalized resistance of the 10-nm-thick samples is reported in Fig. 2b. From this graph it is evident that the films deposited on the $\text {SiO}_2$ insulating substrate show a behavior typical of dirty metals, with a slight increase of the resistance as *T* is lowered, which was also observed on NbRe single crystals^26^. On the other hand, a remarkable decrease of the resistance near room temperature is measured when the Si substrate is used. The effect is more pronounced for the NbRe film 10-nm-thick, which is characterized by a low-temperature resistivity of about $\rho ^{NbRe}_{\mathrm {20K}} \approx$ $240 \pm 20$ $\upmu\Omega$ cm, slightly lower than the one estimated for NbReN, $\rho ^{NbReN}_{\mathrm {20K}} \approx$ $300 \pm 30$ $\upmu \Omega$ cm. We believe that it is reasonable to interpret this effect in the framework of the parallel resistor model^27^. By considering the whole system as a parallel between the film and the substrate, it is clear that the fraction of bias current which flows in the substrate is larger when the film is highly resistive. Moreover, the bare doped Si substrate gets more conductive near room temperature. This suggests that the evident decrease in the *R*(*T*) curve is due to the combination of a high resistivity film with a low resistivity substrate. If a larger resistivity substrate is used, e.g. intrinsic Si, the current sharing between the two parallel resistors will be different, leading to an overall resistance closer to that measured in the case of $\text {SiO}_{{2}}$.

### Voltage-noise properties and fluctuation mechanisms

**Figure 3:**
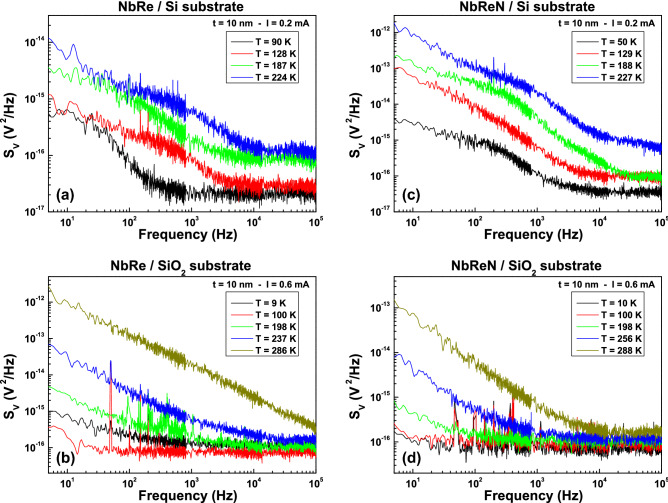
Voltage-noise spectra. Frequency dependence of the voltage-spectral density $S_{V}$ at different temperatures for NbRe (**a**,**b**) and for NbReN (**c**,**d**) films, both deposited on Si and on $\text {SiO}_{{2}}$ substrates. The fixed DC current values are chosen in the bias region where the 1/*f* noise component is characterized by a quadratic current dependence (“linear noise”).

The role played by the substrate and sample thickness in the electrical conduction of NbRe and NbReN films is better understood through noise spectroscopy. This type of experimental investigation is much more sensitive than the measurement of average quantities in visualizing the effects of microscopic motion and transitions^28^. The quantity measured in this kind of analysis is the voltage-spectral density $S_{V}$, whose frequency (*f*) dependence is shown in Fig. 3 for NbRe [panels (a) and (b)] and for NbReN [panels (c) and (d)] ultrathin films ($t = 10$ nm). The presence of a Lorentzian noise component, characterized by a 1/*f*$^{2}$ dependence superimposed to the typical 1/*f* contribution, is clearly evident when the Si substrate is used (upper panels of Fig. 3). This is expected when generation-recombination fluctuation mechanisms induced in conventional semiconductors are the main source of noise^29,30^. Conversely, no sign of such noise process is visible for samples deposited on $\text {SiO}_{{2}}$ substrates (lower panels of Fig. 3), where a pure 1/*f* dependence, characteristic of metallic conductors, both in the clean and dirty limits, dominates^31,32^. Excluding an effect due to superficial defects, which are expected to be negligible, as demonstrated by AFM analysis, the evidence of the Lorentzian bulges is to be attributed to the silicon substrate, whose influence is not only limited to high temperatures, as indicated by DC measurements, but gives a direct contribution also in the low-temperature region, as observed in the noise characterization.

**Figure 4:**
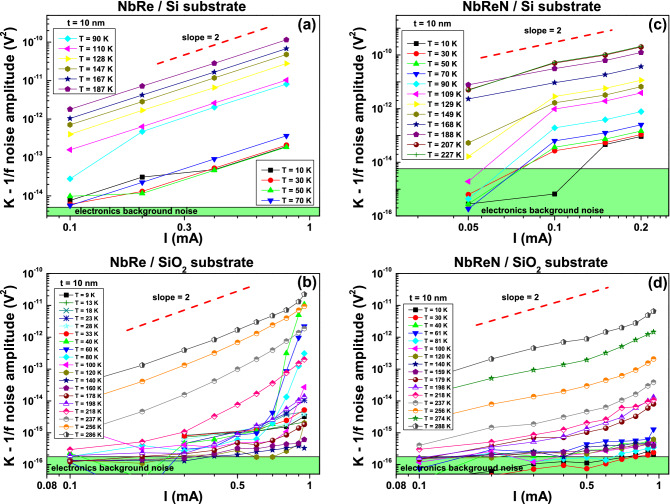
1/*f* noise behavior. Bias current dependence of the 1/*f* noise amplitude *K* in the whole investigated temperature range for NbRe (**a**,**b**) and for NbReN (**c**,**d**) films, both deposited on Si and on $\text {SiO}_{{2}}$ substrates. The green area represents the electronics background noise level.

On the contrary, when analyzing the 1/*f* noise component, a quadratic behavior of its amplitude *K* is found independently from the substrate considered. This type of “linear noise” is indeed present both in the case of Si (upper panels of Fig. 4) and of $\text {SiO}_{{2}}$ (lower panels of Fig. 4). However, the 1/*f* noise component of NbRe films deposited on $\text {SiO}_{{2}}$ substrates shows superlinear power in the current dependence (“nonlinear noise”) in the temperature region below 120 K, namely at high bias current values (see Fig. 4b, for details).

**Figure 5:**
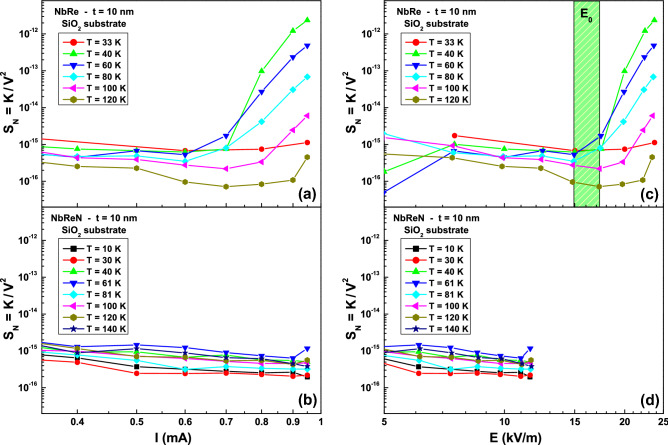
Normalized voltage-noise. The normalized voltage-noise $S_{N}=K/V^{2}$ is shown as function of the bias current *I* (**a**,**b**) and of the locally generated electric field *E* which is parallel to the current flow (**c**,**d**) for NbRe and NbReN 10-nm-thick films grown on $\text {SiO}_{{2}}$ substrates (upper and lower panels, respectively). The green shaded area represents the threshold field interval $E_{0}$.

To shed a light on this behavior it is useful to introduce the normalized voltage-spectral density, $S_{N}=K/V^{2}$, where *V* is the measured DC voltage. This quantity can be analyzed as a function of both the bias current, *I*, see panels (a) and (b) in Fig. 5, and the local electric field, *E*, see panels (c) and (d) in Fig. 5. Indeed, while no scaling is found in the $S_{N}$ versus *I* dependence either for NbRe and for NbReN films [panels (a) and (b) of Fig. 5], a clear threshold between two distinct behaviors is evident in the $S_{N}$ versus *E* graph for the NbRe film. In particular, below a threshold field range $E_{0}$ [the green shaded region in Fig. 5c ranging from 15 to 17 kV/m], the independence of $S_{N}$ from *E* is the signature of a “linear noise” component. Conversely, above $E_{0}$ the evident increase of $S_{N}$ with *E* indicates the occurrence of “nonlinear” fluctuations. This experimental finding has been already observed in iron-based superconductors^33^, and it is typically related to inhomogeneous composites^34^. Following the proposed theoretical framework, it is suggested that, above a threshold field, the noise is caused by fluctuations associated to thermally activated transitions between metastable states^34^. In the case of the samples considered here, the nature of such metastable states is not clear and should be further investigated. In different systems, an origin due to the existence of charge density waves has been reported^35–37^. Unfortunately, this dependence cannot be confirmed for NbReN ultrathin films, because the measurement setup limited the range of obtainable electric field.

**Figure 6:**
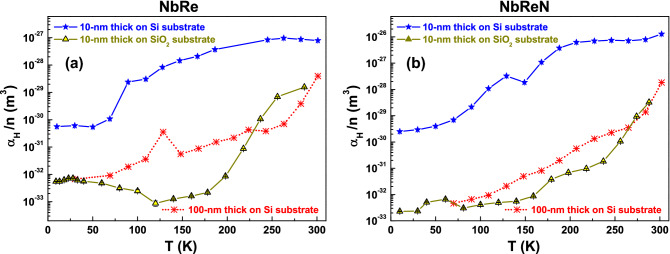
The semi-empirical Hooge parameter. The temperature dependence of the normalized Hooge parameter $\alpha _{H}/n$, extracted from Eq. (1), is shown for NbRe (**a**) and for NbReN (**b**) ultrathin films grown on Si substrates (blue stars) and on $\text {SiO}_{{2}}$ substrates (yellow triangles). The $\alpha _{H}/n$ versus *T* dependence for 100-nm-thick films is also reported for comparison with red dotted lines and asterisks for films of both NbRe (**a**) and NbReN (**b**).

More information, mostly from the point of view of applications, can be extracted by studying the “linear noise” component. In this regime, the strictly quadratic current dependence of the 1/*f* noise allows one to define the normalized Hooge parameter as^31,38^

1 $$\begin{aligned} \frac{\alpha _{H}}{n}\left( T\right) =\Omega f S_{N}\left( T\right) ~, \end{aligned}$$

where $\Omega$ is the sample volume. The Hooge formula of Eq. (1) has no general physical explanation, but is used to compare the noise level in different materials of different size. The temperature dependence of $\alpha _{H}/n$ is shown in Fig. 6a and in b for NbRe and NbReN ultrathin films, respectively. It is evident a clear reduction (more than two orders of magnitude) of the intrinsic noise level when the insulating $\text {SiO}_{{2}}$ substrate (yellow triangles) is used with respect to Si (blue stars) and, even more interesting, the very low noise observed for NbRe and NbReN is comparable or even smaller than the one found for NbN^39–41^. It is worth to underline that at low temperatures NbReN is characterized by noise levels almost one order of magnitude lower than NbRe. The comparison of the noise behavior of films of different thickness [see red dotted lines and asterisks in panels (a) and (b) of Fig. 6] shows a much lower noise level in the case of 100-nm film on Si substrate, when compared with the 10-nm film on the same substrate. Such noise level is comparable with that of the 10-nm film on $\text {SiO}_{{2}}$, suggesting that the observed noise is closer to the intrinsic material value. Therefore, it is possible to conclude that, for the 10-nm-thick film on Si substrate, the generated noise is dominated by the substrate noise generation processes. We note that, in the case of NbRe on $\text {SiO}_{{2}}$, the $\alpha _{H}/n$ value has a minimum at about $T = 120$ K, and than, by lowering the temperature, raises towards a constant value. This is different with the behavior of NbReN noise, which is characterized by a continuous decrease with temperature. The reasons for the low-temperature behavior of the ultrathin NbRe films is at present unknown. The low noise levels, also at reduced sample thickness, indicate a good film quality as far as electrical conduction is concerned. This is a mandatory requirement for the possible application of both NbRe and NbReN films in the field of low-temperature electronics.

## Conclusions

NbRe and NbReN films 10- and 100-nm-thick have been deposited on Si and $\text {SiO}_{{2}}$ substrates, whose choice does not seem to strongly influence the morphological and superconducting properties. On the contrary, as far as the electronic transport is concerned, the Si substrate affects the DC normal state electrical transport properties only in the high-temperature region above 200 K, while the charge carrier fluctuation mechanisms are influenced by the substrate in the whole *T* range. More in details, the films grown on $\text {SiO}_{{2}}$ are characterized by lower values of the intrinsic noise than the ones deposited on Si. This result must guide the choice of the proper substrate to use in view of the possible development of SNSPDs based on these superconducting materials. In this respect, the use of an insulating substrate, such as $\text {SiO}_{{2}}$, seems to be more appropriate since it gives a direct contribution for the reduction of additional electronic noise sources. In particular, very low noise amplitude values are measured both in ultrathin and in 100-nm-thick films of both superconductors. NbReN films, moreover, are characterized by smaller noise levels compared to NbRe ones at low temperatures. These experimental findings confirm the potential of NbRe-based films for the future application in low-temperature electronic devices.

## Methods

### Sample preparation

$\text {Nb}_{{x}}\text {Re}_{1-x}$ (namely, NbRe) and (NbRe)$_{1-x}\text {N}_{{x}}$ (namely, NbReN) films were deposited from a $\text {Nb}_{0.18}\text {Re}_{0.82}$ 99.95% pure target of 5 cm in diameter by ultrahigh vacuum DC diode magnetron sputtering at room temperature. The system base pressure is in the low $10^{-8}$ Torr. NbRe films were sputtered in an Ar pressure of $P_{Ar}= 4 \times 10^{-3}$ mbar at a deposition rate of about 0.40 nm/s. The Nb concentration, as estimated by energy diffraction spectroscopy (EDS), was $x = 0.18$. X-ray diffraction (XRD) performed in Ref.^6^ confirmed that the films grow in a noncentrosymmetric $\alpha$-Mn cubic phase^42,43^. The NbReN films were reactively sputtered in a mixture of Ar and $\text {N}_{{2}}$ gases, with a total pressure during the deposition of $P_{tot}= 4 \times 10^{-3}$ mbar and a $\text {N}_{{2}}$ percentage of 25% of $P_{tot}$, which results in a deposition rate of 0.15 nm/s and in an atomic concentration ratio measured by EDS of about $\text {N}_{{2}}$/NbRe $= 1.0 \pm 0.2$^8^. XRD spectra acquired in Ref.^8^ suggest that the films are polycrystalline, but at this stage no details of the unit cell are available. Some of the investigated samples were patterned by standard optical lithography (lift-off) into a Hall bar geometry of width $W = 10$ $\upmu$m and distance between the voltage contacts $L = 90$ $\upmu$m. The van der Pauw technique^44^ was used to determine the resistivity of the unpatterned films. Two different film thicknesses, $t = 10$ and $t = 100$ nm, are considered in this work. They correspond, respectively, to the ultrathin regime, appealing for SNSPDs applications, where *t* is comparable to the superconducting coherence lenght $\xi$^6^, and to the limit of large thickness, where bulk properties of the materials are well established. The influence of the substrate on the transport processes of the films is investigated by using two different substrates, namely Boron-doped Si(100) and Boron-doped Si(100) covered by a 200-nm-thick amorphous $\text {SiO}_{{2}}$ layer.

### Morphological measurements

 The used AFM system is a Nanite by Nanosurf equipped with a Tap190Al-G monolithic silicon Tip (Budget Sensors). The measurements have been performed in air in tapping mode with a 48 N/m constant force and 190 kHz resonance frequency. AFM images have a 0.2 nm rms vertical resolution and a 1.5 nm rms lateral resolution.

### Electrical transport and noise measurements

 Resistance versus temperature, *R*(*T*), measurements were performed in a liquid $^4$He cryostat by using a custom-made dip probe by using a standard four-wire configuration. The samples were biased by a DC current between 10 and 50 $\upmu$A provided by a Keithley 2400 current source, while the voltage drop was measured by a Keithley 2182A nanovoltmeter. The AC voltage-noise measurements were performed in a standard four-probe configuration, with contacts ultrasonically bonded to the pads realized by optical lithography. The investigated temperature range, from 300 down to 10 K, was varied by using a closed-cycle refrigerator system (Janis Research) with an active temperature stabilization of 0.2 K by means of a Proportional-Integral-Derivative (PID) algorithm. The sample bias was made with a Keithley 220 DC current source, while the DC voltage signal was acquired with a Keithley 2002 nanovoltmeter. The AC signal, instead, was amplified with a home-made electronics optimized for low-noise measurements^45^, and was analyzed with a HP35670A dynamic signal analyzer, by accurately reducing external spurious and contact noise contributions^46^. The unwanted effect due to Joule heating was also ruled out, as demonstrated in supplementary Fig. S2 where two *R* versus *T* curves, taken with bias current values of 100 μA and 2 μA, do not show any difference.

## Supplementary Information

Supplementary Figures.
