# Supplementary material for: Effect of the substrate on the electrical transport and fluctuation processes in NbRe and NbReN ultrathin films for superconducting electronics applications
Source: Sci Rep. 2022 Jan 28;12:1573. doi: 10.1038/s41598-022-05511-5 (PMC8799732; doi:10.1038/s41598-022-05511-5)
Supplement: Supplementary file 1 — Supplementary Figures. [file 41598_2022_5511_MOESM1_ESM.pdf]

# Supplementary Information for "Effect of the substrate on the electrical transport and fluctuation processes in NbRe and NbReN ultrathin films for superconducting electronics applications"

C. Barone[1,2,3] *, C. Cirillo[2,1], G. Carapella[1,2,3], V. Granata[1,2], D. Santoro[1], C. Attanasio[1,2,3], and S. Pagano[1,2,3]

[1]*Dipartimento di Fisica "E.R. Caianiello", Università degli Studi di Salerno, I-84084 Fisciano, Salerno, Italy.*

[2]*CNR-SPIN, c/o Università degli Studi di Salerno, I-84084 Fisciano, Salerno, Italy.*

[3]*INFN Gruppo Collegato di Salerno, c/o Università degli Studi di Salerno, I-84084 Fisciano, Salerno, Italy.*

(* email: cbarone@unisa.it)

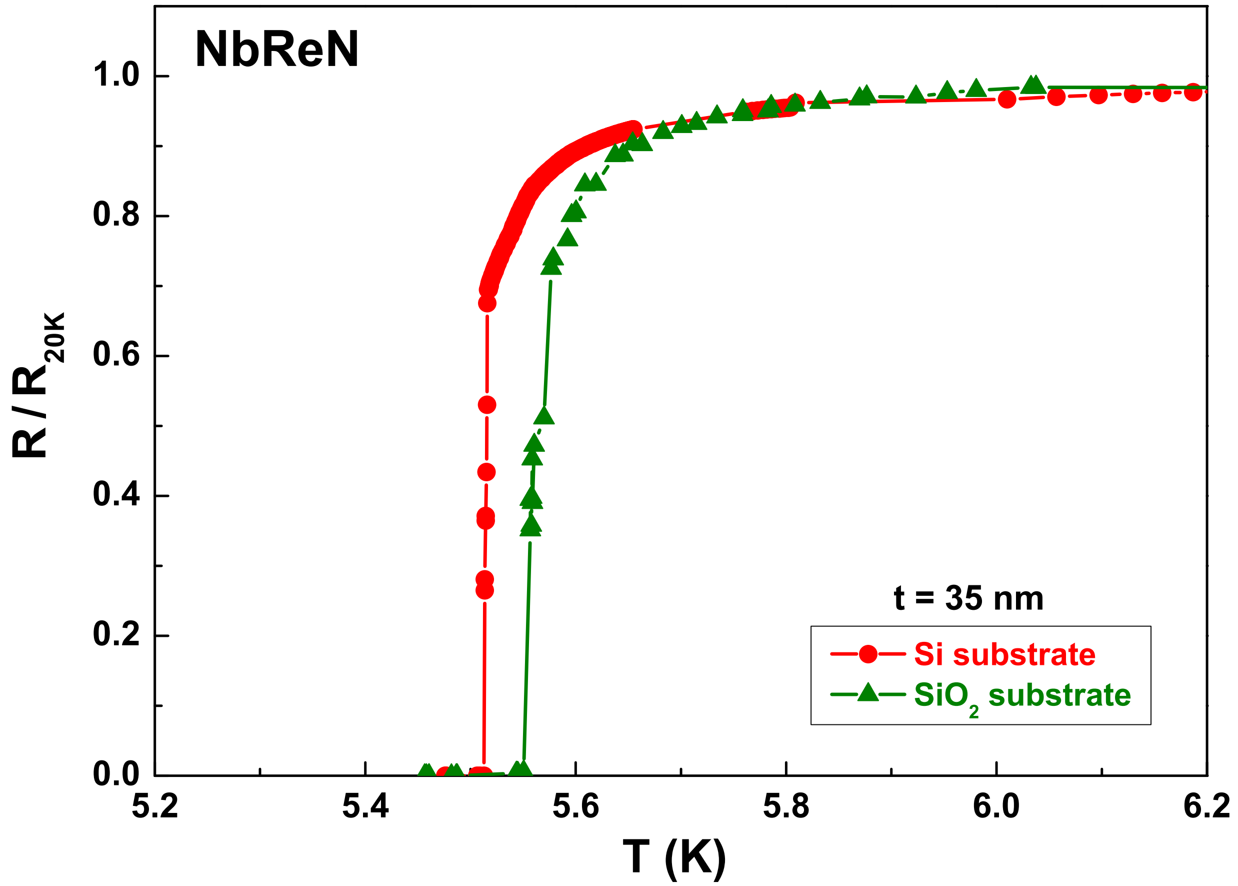


Fig. S1. **Normalized resistance near the superconducting transition.** Normalized resistive transitions, $R/R_{20\mathrm{K}}$, for two NbReN 35-nm-thick films fabricated in the same deposition run on both Si (red circles) and $SiO_2$ (green triangles) substrates.

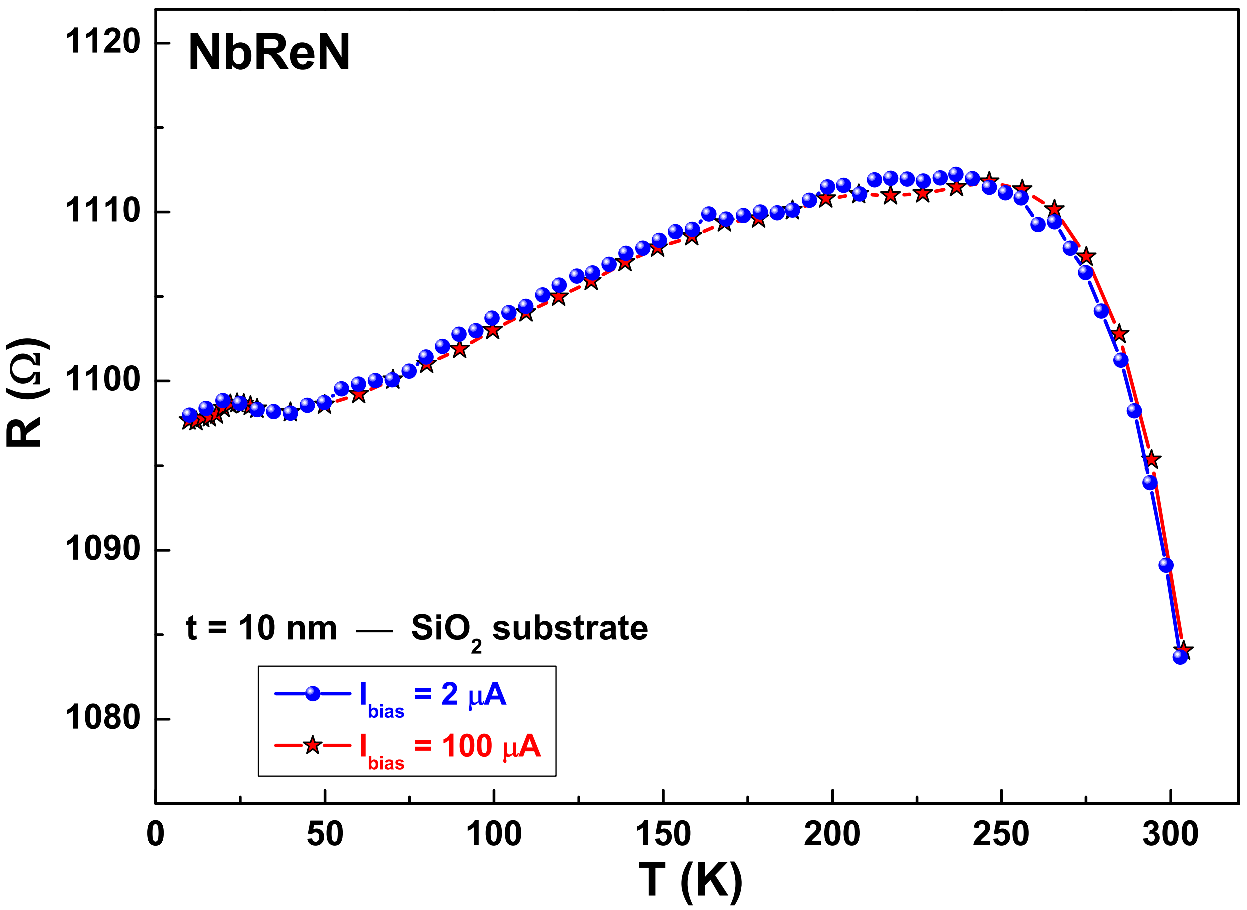


Fig. S2. $R$ **versus** $T$ **curves at different bias currents.** The temperature dependence of the resistance is shown for a typical NbReN 10-nm-thick film deposited on $SiO_2$ substrate at two different bias current values: 2 $\mu$A blue dots and 100 $\mu$A red stars. No differences are clearly evidenced, ruling out the possible presence of Joule heating effects.
